# Open-Air Wards in Peace and War

**Published:** 1915-07-17

**Authors:** 


### OPEN-AIR WARDS IN PEACE AND WAR.

### The Lessons of the Cambridge Experiment.

The treatment of disease in the open air, save for certain surgical cases, has for one reason or another not received any great encouragement in recent years, though its possibilities and advantages have been recognised as the result of trials on comparatively small scales in various institutions. The sanatorium treatment of tuberculosis is, of course, in another category. It is, perhaps, in connection with the treatment of patients suffering from measles, scarlet fever, and other infectious fevers that one might express some surprise that "open-air wards" have not been more generally advocated. The results obtained in such diseases are, we believe, satisfactory, and markedly effective in reducing the incidence of complications, and the length of stay of the patients in hospital. Even such a limited application of the principle as is possible in the London Fever Hospital at Islington has proved the benefit of treating the broncho-pneumonia of measles in the open air.

### A Cambridge Pamphleteer.

Public interest in the question of open-air hospitals has lately been greatly awakened by the success of the 1st Eastern General Hospital at Cambridge. This interest has been stimulated in no inconsiderable degree by the indefatigable Dr. Shipley, who did much to focus attention on the insect carriers of disease in his small book, which was reviewed in The Hospital of July 3, entitled, "The Minor Horrors of War." The Master of Christ's College, Cambridge, has, naturally, a close local interest in the work of so large a hospital as that which has been erected on one of the best cricket pitches in his University. He, therefore, soon obtained the hospitality of the columns of *Country Life*, and contributed several enthusiastic articles on the construction and working of this great military hospital. These articles have now been republished in a well-printed, illustrated pamphlet called "The Open-Air Treatment of the Wounded."* From these pages the public may gather a good general idea of an "open-air" hospital on a large scale.

From the point of view of the hospital expert, the pamphlet leaves something to be desired, and it is, perhaps, unfortunate that Dr. Shipley had not the advantage of a professional collaborator in compiling his entertaining pages. But he has certainly achieved the aim of the articles; that is, the convincing of the public that in this climate, even at some 30 feet above sea level, which, if we remember, Cambridge is, the possibilities of the so-called open-air hospital are practically unlimited, and the advantages substantial. Dr. Shipley's general remarks are written characteristically, and we can but congratulate him on the charm and conviction of his advocacy. Our readers will accept his argument, and will turn more readily to those practical details which are provided.

* Country Life Library. Price 1s.

### Two Practical Points.

The hospital contains, as readers of The Hospital may remember, ten long blocks of buildings running east and west, the open side naturally facing south. Over twelve hundred beds are provided. The wooden framework has walls covered externally with asbestos slabs, resting on brick foundations with a bitumen damp-proof course. The roof is covered with "ruberoid" weatherproof sheeting which is largely fire-resisting. On the south side of the wards protection against rain is afforded by blinds, which apparently have not been found free from serious defects. It is easy to imagine that, as Dr. Shipley says, "they flap and flop and cause an irregular and intermittent noise at night." The irregularity of the noise is undoubtedly the chief evil. Under the heading "The Kitchen," some interesting details are given with which most hospital workers will be familiar. A practical hint is given in connection with the peeling of potatoes. A central ring only of peel is removed, leaving a large cap at each end of the potato. This is aptly described as cooking potatoes "in their jackets, but with the belt off." It is not stated how this manœuvre is executed, but the implication is that it is done by hand and not by machinery.

### £16 Per Bed, and Patients' Length of Stay.

The results achieved in the hospital have already been made known, and it is sufficient to recall the fact that the very low mortality figures are not to be ascribed to the absence of bad cases. The cost per bed of this hospital has been estimated at about £16. To compare fairly this cost with the £300 or £400 which is often the cost per bed in London hospitals some further details would be necessary, but it is sufficiently obvious that an open-air hospital on these lines can be erected and managed economically.

A point is made of the saving of time to the patients, who, on discharge, are so much more fit than those discharged from an ordinary hospital that the usual two or three weeks' stay in a convalescent home becomes an unnecessary addition to their treatment. This point is one which cannot fairly be thrown into the scale against hospitals in the midst of large populous districts.

### The Idea and its Executors.

The scheme of this hospital was thought out many years ago by Dr. Joseph Griffiths, and he was ably seconded by experts almost entirely drawn from the town of Cambridge. The architect, Mr. C. F. Skipper; the builders, Messrs. A. Negus and Son; the electric-light installers, and the gas engineers are all credited to the University town, but the heating installation was provided by Messrs. F. A. Norris and Co., of London.

Of the two other chapters in this pamphlet one is devoted to the staff, and is headed by an unexpected if rather strained excerpt from the 23rd Psalm, which makes us wonder whether the Psalmist or the author's University colleague, Mr. A. C. Benson, really attracted Dr. Shipley's attention to it. The other chapter is by Dr. W. J. Simpson, and refers to open-air treatment in general, with instances of highly successful past experiments in the treatment of disease under open-air conditions. Several of these trials, by the way, were accidental and quite unintentional.

Other Examples of Open-Air Hospitals.

The public seem to imagine that the construction of open-air wards is a much simpler matter than it really is, and that the 1st Eastern General Hospital is the embodiment of a new principle in hospital construction. It is therefore advisable to draw attention to the fact that open-air wards, as distinct from balconies and *Liege Halle*, have been in use in this country for some years; not, it is true, on so large a scale as at Cambridge, but still on more than an experimental basis. Perhaps one of the best instances to quote is that of the Royal Liverpool Country Hospital for Children at Heswall. This hospital was reconstructed in 1908, and now accommodates about 150 children in three-sided wards. The success attending this institution has been largely due to the efforts of Dr. Macalister and Mr. Robert Jones in advocating the advantages of fresh air. Considerable accommodation of this nature also exists at the Queen Mary's Hospital for Children at Carshalton, which was very fully described in The Hospital of July 26, 1913, page 507.

The Staff's Difficulty.

The nursing staff do not regard open-air wards as an unmixed blessing, for the nurses do certainly feel the cold, especially those on night duty. This fact will be minimised by experience and use, but its existence at all does not indicate wisdom or foresight on the part of the authorities. It is an administrative duty to see that the nursing staff are suitably clothed so as to remove all cause for complaint. The patients are not to be pitied, for they have extra blankets and hot-water bottles when necessary, while nurses must rely on work and movement to keep warm—a difficult matter at night when things are quiet. But, as one of the nurses said to Dr. Shipley, "The worst of it is we have never been so healthy in our lives, so it is difficult for us to complain."

Dr. Shipley's pamphlet may be commended to the notice of all institutional workers, and will doubtless be treasured as a souvenir by the friends and relations of the large numbers of soldiers who have passed through its airy wards. In this way it should at once act as an educational volume and, if we may say so, as an appeal. Few documents combine both these characteristics. There are a few places in which one is surprised by loosely constructed sentences such as a master of a University College would hardly pass in his capacity as examiner; yet the tone of the descriptive text is stimulating and convincing enough for any pseudodoxia or "vulgar errors" to be forgiven, even in that very important revelation of clear-headedness or muddle-headedness which we call style, or want of style, in literature.

